# Relation of increased BMI to left atrial volume in repaired tetralogy of Fallot patients

**DOI:** 10.1186/1532-429X-18-S1-P172

**Published:** 2016-01-27

**Authors:** Jason Christensen, Scott Simpson, Suzanne L Field, David Parra, Jonathan H Soslow

**Affiliations:** 1 Pediatrics, Vanderbilt Medical Center, Nashville, TN USA; 2 grid.410721.10000000419370407Pediatric Cardiology, University of Mississippi Medical Center, Jackson, MS USA; 3 grid.412807.80000000419369916Pediatric Cardiology, Vanderbilt Medical Center, Nashville, TN USA

## Background

Contemporary care of repaired tetralogy of Fallot (rTOF) patients is directed toward detection and avoidance of long-term morbidity and mortality, of which arrhythmias play an important role. Increased arrhythmia risk in the rTOF population has been associated with ventricular dysfunction and chamber dilation. In adults without congenital heart disease, obesity has been related to left atrium (LA) dilation and further associated to arrhythmias. The effect of obesity on LA size, and in turn on arrhythmia, in rTOF patients has not been studied. If related, obesity would represent a modifiable risk factor in this population. We hypothesized that LA dilation is associated with obesity in rTOF patients.

## Methods

Patients with rTOF undergoing cardiac magnetic resonance imaging (CMR) performed from 2009-2013 were retrospectively reviewed. Patients were excluded for inadequate image quality and mitral regurgitation graded worse than mild by echocardiography performed within a year of CMR. Left and right ventricular (LV and RV) size and ejection fraction (EF) were measured. LA size was calculated by Simpson's biplane formula using LA area and length in the 2- and 4-chamber views. Body mass index (BMI) was calculated on all patients and dichotomized by BMI percentiles into two categories: normal and overweight/obese. Univariate associations were evaluated using Spearman correlation coefficients for continuous variables and Mann-Whitney U test for dichotomous variables.

## Results

73 patients were included with a mean age at CMR of 20.2 years (range: 5.7-54.8); 57.5% were female. Average age at repair was 15.8 months. Mean BMI was 23.8 kg/m2. 57.5% had normal weight and 42.5% were overweight/obese. Only 7 patients had confirmed arrhythmia, likely due to the young age of the cohort. Mean indexed RV end-diastolic volume was 140 ml/m2 and RVEF was 52.7%. Mean indexed LV end diastolic volume was 64 mL/m2, and mean LVEF was 63.4%. Mean absolute and indexed LA volumes were 43.2 ml ± 14.0 and 29.7 ml/m2 ± 13.9, respectively. The correlations between absolute and indexed LA volume with BMI were 0.435 and 0.483 (p < 0.001 for both). Patients in the overweight/obese category had significantly increased absolute and indexed LA volume compared with those in the normal weight category (47.3 ml ± 13.4 vs 40.3 ml ± 14.0 and 32.7 ml/m2 ± 13.2 vs 27.5 ml/m2 ± 14.0, p = 0.020 and p = 0.023, respectively). Patients with arrhythmia had increased absolute LA volumes and a trend towards increased indexed LA volumes (53.9 ml ± 13.4 vs 42.0 ml ± 13.7 and 33.2 ml/m2 ± 2.9 vs 29.3 ml/m2 ± 14.5, p = 0.03 and p = 0.064, respectively).

## Conclusions

Elevated BMI is associated with LA dilation in patients with TOF, which may put these patients at risk for negative cardiovascular outcomes including arrhythmia. Further study is warranted in investigating the complex interaction between obesity and clinical outcomes in congenital heart disease.

**Figure 1:**
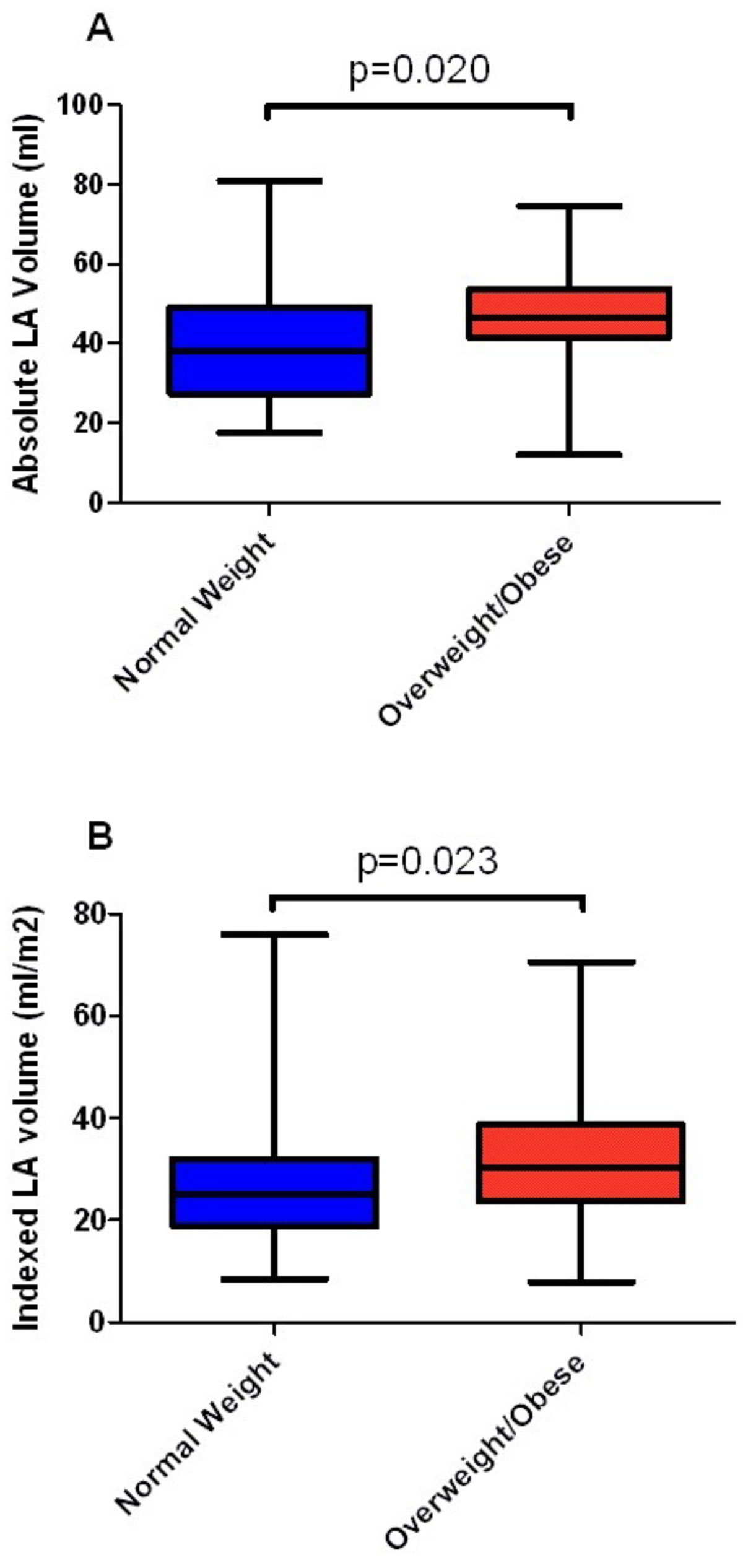
**Absolute and indexed LA volume vs. BMI category**.

